# Immune Checkpoint Inhibitors to Treat Malignant Lymphomas

**DOI:** 10.1155/2018/1982423

**Published:** 2018-04-11

**Authors:** Magdalena Witkowska, Piotr Smolewski

**Affiliations:** Department of Experimental Hematology, Medical University of Lodz, Lodz, Poland

## Abstract

Genetic and/or epigenetic changes provide antigen-derived diversity in neoplastic cells. Beside, these cells do not initiate immune response of host organisms. A variety of factors are responsible for the resistant to treatment, including individual variations in patients and somatic cell genetic differences in tumors, even those from the same tissue of origin. Immune system is controlled by several controlling mechanisms. Recently, a significant progress in hematologic treatment has been made; however, majority of diseases still remain incurable. Immunotherapy with checkpoint inhibitors has emerged as promising modality of antitumor treatment, showing marked response to several antigens, including cytotoxic T lymphocyte-associate protein-4 (CTLA-4) or programmed cell death 1 receptor (PD-1). In this review, we demonstrate actual knowledge on immune checkpoint function and its impact on development of new modality of antineoplastic treatment, using, for example, anti-CTLA-4 or PD-1/PD1 ligand (PD-L1) monoclonal antibodies in malignant lymphomas.

## 1. Introduction

Immune checkpoint blockade therapy gained significant success in treatment of several refractory solid tumors. A new generation of cancer treatment strategy called targeted therapy has been developed for the treatment of many hematological cancers including Hodgkin lymphoma (HL), diffuse large B cell lymphoma (DLBCL), or follicular lymphoma (FL). A number of novel immunological drugs targeting tumor microenvironment have been developed. Unfortunately, some lymphoma patients are not eligible for targeted therapies and not all patients receiving targeted agents actually respond to it. Furthermore, conventional chemotherapy causes wide range of toxicities including bone marrow suppression. The immune system is crucial for identifying and destroying “foreign” cells, such as cancer cells. Tumor cells, however, use certain strategies to avoid recognition by the immune system, so as to grow unchecked [1]. Among these, the one strategy that is most credulous in the activation of a counterattack is “immune checkpoint activation.” The most valuable seems to be checkpoint inhibitors represented by two main pathways: antiprogrammed cell death 1 receptor (PD-1) antibodies, such as nivolumab and pembrolizumab, and antibody directed against cytotoxic T lymphocyte-associate protein-4 (CTLA-4), such as ipilimumab. The mode of these agents' action is to release the brakes that block the action of the immune system against the tumor [2]. A great impact of checkpoint inhibitors on cancer immunotherapy was observed especially for melanoma, non-small-cell lung cancer, and renal-cell carcinoma. Recent clinical studies have illustrated promising outcomes in HL for these drugs used as single agents and in combination with traditional therapeutics. In this article, the authors discuss therapy with checkpoint inhibitors mainly in HL patients as they are becoming integrated into treatment paradigms.

HL is a rare clonal disease of the lymphatic system that arises from B cells of germinal and postgerminal centers. The frequency of HL in Western European countries is around 10% of lymphoma types and approximately 0.5% of all neoplastic disease [3]. Based on differences in the neoplastic cell phenotype and the histological picture, HL is divided into two subtypes: classical HL (cHL) and nodular lymphocyte-predominant HL. cHL is diagnosed in majority of patients in approximately 95%. Characteristic for all subtypes of cHL is the presence of neoplastic Reed-Sternberg (RS) cells, which are not observed in any other malignant diseases [4]. RS cells are in minority, while the tumor in majority is composed of an inflammatory background, crucial for growth and survival of cancer cells. Lymphoma microenvironment is composed of various cell types including the most common histiocytes, eosinophils, lymphocytes, and plasma cells. They are responsible for interacting with numerous cells including CD4+ and CD8+ T lymphocytes, B lymphocytes, plasma cells, or dendritic cells, through secretion of different chemokines and cytokines [5]. The complex microenvironment interactions are unique among lymphomas and are responsible for initiation and progression of HL.

Nowadays, HL is a highly curable cancer with long-term survival exceeding 85%, but still about 15% of patients have progression after first-line chemotherapy. Moreover, there are still approximately 30% of patients who will relapse after front-line treatment [6]. Generally, successful treatment of HL is connected with long-term adverse events (AEs). HL survivors might present years after treatment-related complications such as secondary neoplastic disease, lung fibrosis, cardiovascular disease, and hypothyroidism.

On another hand, in resistant/relapsed (R/R) HL patients who are eligible for transplantation, high-dose chemotherapy and autologous stem-cell transplantation (ASCT) is currently a standard of care. Unfortunately, prognosis for those groups is rather poor with possibility to achieve a complete remission (CR) in less than 50% with a median overall survival (OS) of approximately 2 years [7]. Allogeneic hematopoietic stem-cell transplantation (allo-HSCT) is also a therapeutic option in R/R HL cases, the chance for long-term remission.

For several years, treatment options for patients with HL were limited to combination of chemotherapies and radiotherapy. Better understanding of the pathobiology and signaling pathways in tumor microenvironment effected in the development of novel agents and improved results of the therapy and extended the quality of life.

A separate group of lymphoproliferative malignancies are non-Hodgkin lymphomas (NHLs). NHLs represent a heterogeneous group of tumors of different biology, prognosis, etiopathogenesis and epidemiology. Particular types of NHLs differ also in regard to morphology and possess various immunophenotypic, genetic, and clinical features. Some of them are still incurable, despite introducing novel treatment strategies, such as immunotherapy or targeted, biological treatment (indolent lymphomas, including FL). In contrast, different types of NHLs, with more aggressive course, such as DLBCL or Burkitt lymphoma, may be cured in high percent of patients with intensive immunochemotherapy. However, still there is significant proportion of R/R patients, who require rescue with new, more specific treatments.

## 2. Immunobiology of Checkpoint Blockade

Over the last decade, a deeper understanding of immune regulatory pathways and the immunophysiologic interactions in tumor microenvironment effected in the invention of novel drugs in the treatment of different malignant diseases, including HL. Recently, immune checkpoint inhibitors have become a new promising way of immunotherapy. The strategy to reduce inhibitory signaling and restore the patients' natural tumor-specific T cell-mediated immune responses is now being developed.

Cancer is ultimately the result of uncontrollable cell growth and is often seen in patients with impaired immunity. Tumor antigen presentation to T cells and T cell activation lead to death of cancer cell. Immune response is initiated through antigen recognition by the T cell receptor. Its amplitude and quality are regulated by influence between costimulatory and inhibitory signals that are immune checkpoints. As this coinhibition is responsible for suppressing autoimmunity, the expression of immune checkpoints may be dysregulated by malignant disease and allow cancer cells to “escape” from the immune system [2]. Several stimulatory checkpoint molecules are members of the tumor necrosis factor receptor (TNFR) superfamily: CD40, CD27, CD137, OX40, and glucocorticoid-induced TNFR family-related gene (GITR). Expression of CD40 was found on a variety of immune cells including antigen-presenting cells (APCs) that have CD40 ligand transiently expressed on activated CD4+ cells [8]. CD27 molecule supports antigen-specific expansion of naïve T cells and inhibits function of Th17 effector cells [9, 10]. In turn, CD137 molecule-mediated signaling protects T cells and, in particular, CD8+ cells from activation-induced apoptosis [11]. The molecule called OX40 (CD134) not only promotes the expansion of effector and memory T cells but can also suppress differentiation and activity of T regulatory cells (Tregs) [12]. GITR stimulates expansion of T cells, including Tregs, and its ligand is expressed on APCs [13].

Another stimulatory molecules that belong to the B7-CD28 superfamily are CD28 and inducible costimulatory T cell (ICOS; CD278). CD28 antigen is constitutively expressed on CD4+ cells and on some CD8+ cells. Its ligands are expressed on dendritic cells, CD80, and CD86, stimulating proliferation of T cells [14]. Finally, ICOS expressed on activated T cells is a molecule important for T cell effector functions [15].

Among the group of checkpoint inhibitors, there are many different molecules including adenosine A2A receptor (A2AR), lymphocyte activation gene-3 (LAG-3), indoleamine 2,3-dioxygenase (IDO), B and T lymphocyte attenuator (BTLA), MHC class I killer-cell immunoglobulin-like receptor (KIR), mucin domain 3 (TIM-3), and V-domain Ig suppressor of T cell activation (VISTA) [16–24]. However, the most significant seems to be programmed death 1 (PD-1) receptor and CTLA-4 [25, 26].

Several mechanisms contribute to cancer cell escape from the immune response, including decrease in expression of the MHC class I molecules on tumor cells, preventing their recognition by CTLs. Importantly, cancer cells lost their immunogenic antigens. In addition, growing cancer acquires immunological tolerance, by having mutated proteins and altered antigen expression, which prevent elimination by the host immune system. Tumor microenvironment among population of mutated cells is composed of different types of cells including stromal cells, macrophages, blood vessels, immune cells, fibroblasts, bone marrow-derived inflammatory cells, lymphocytes, signaling molecules, and the extracellular matrix (ECM). These cells and their interactions all contribute to the changing tumor microenvironment, which the tumor largely manipulates to be immunotolerant so as to avoid elimination. There is an accumulation of metabolic enzymes that suppress T cell proliferation and activation, including IDO and arginase, and high expression of tolerance-inducing ligands like FasL, PD-1, CTLA-4, and B7. Finally, the important mechanism of tumor cell immunoescape is abnormal polarization of tumor-associated macrophages inducing PD-1 expression and promoting the development of Tregs [27–29].

Immunosuppression is partly mediated by PD-1 and CTLA-4, two immunomodulatory receptors expressed on T cells that trigger inhibitory pathways dampening T cell activity. The interaction between the receptor PD-1 (expressed on T cells and on other immune cells of the inflamed tumor microenvironment) and its ligands PD-L1/L2 (expressed on myeloid dendritic cells, activated T cells, some nonhematopoietic tissues, and tumor cells) determines a downregulation of T cell effector functions. This event minimizes the tissue damages, prevents the development of autoimmunity through the promotion of tolerance to self-antigens, and, in cancer patients, inhibits the antitumor immune response [30].

**Figure 1:**
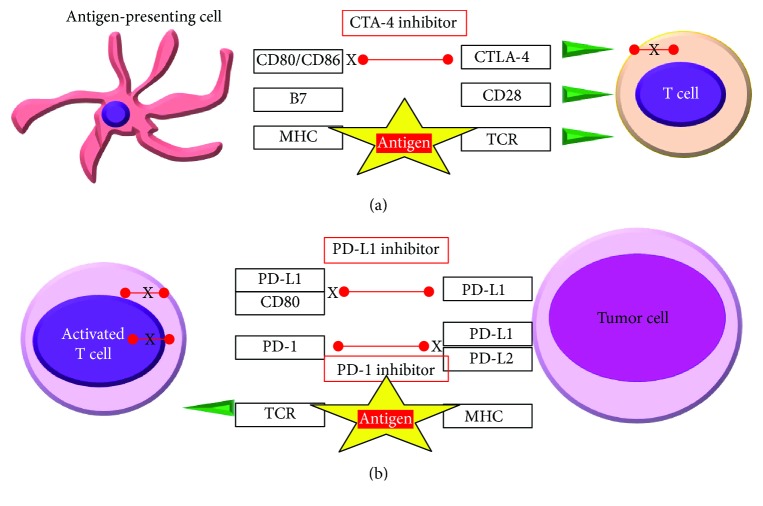
Interactions between activated T lymphocytes and tumor by the CTLA-4 (a) and the PD-1 pathway (b). APC: antigen-presenting cell; CTLA-4: cytotoxic T lymphocyte-associated protein 4; MHC: major histocompatibility complex; PD-1: programmed cell death 1; PD-L1: programmed cell death ligand 1; TCR: T cell receptor.

Thus, the interaction of the PD-1 on activated T cells with its ligands PD-L1 and PD-L2 remains immunologic tolerance through the suppression of autoreactive T lymphocytes. On the other hand, CTLA-4 is a coinhibitory receptor on T cells that may be responsible for inducing T cell tolerance [31]. CTLA-4 is expressed on recently activated T CD4+ and CD8+ lymphocytes. The expression of CTLA-4 on Treg cells serves to control T cell proliferation [26]. It inhibits T cell activation upon binding with B7-1 and B7-2 (costimulatory molecules present on APC surface), counteracting CD28-mediated signals. Still, the role of CTLA-4 in dampening T cell activity is not fully understood, while several other mechanisms have been proposed such as extrinsic effect which do not depend on the signaling properties of CTLA-4. This process can be reversed with a treatment that inhibits immune checkpoints and intensifies endogenous anticancer immune response cells. When these proteins are blocked, the “brakes” on the immune system are released and T cells are able to kill tumor cells properly (Figure 1). Examples of checkpoint proteins found on T cells or cancer cells include PD-1/PD-L1 and CTLA-4/B7-1/B7-2.

Several immune checkpoint inhibitors targeting PD-1 or CTLA-4 have been developed (Table 1). So far, the most valuable seems to be checkpoint inhibitors represented by anti-PD-1 antibodies such as nivolumab and pembrolizumab and anti-CTLA-4 antibody such as ipilimumab. There are evidences of successful treatment of melanoma, lung cancer, and renal cell carcinoma, what in turn led to clinical U.S. Food and Drug Administration (FDA) approval for ipilimumab (2011) and nivolumab or pembrolizumab (both in 2014).

## 3. The Expression and Role of PD-1 and CTLA-4 Pathway in Lymphomas

PD-L1/PDL-2 overexpression has been already reported in hematological malignancies. The expression of PD-L1 on tumor cells has been found to be regulated by several cytokines, including interferon gamma produced by T cells from tumor microenvironment [32]. PD-L2 expression is present in lymphomas with abnormalities in 9p24.1/PD-L1/PD-L2. The only exception may be DLBCL, in which PD-L2 expression of RNA and protein is not connected with cytogenetic abnormalities in 9p24.1. Moreover, in cHL, the amplification of 9p23-24 gene is responsible for PD-L1/PDL-2 encoding in RS cells, as well as for the activation of Janus kinase 2 (JAK-2) gene, what additionally increases production of PD-L1 through JAK/signal transducer activator of transcription (STAT) signal transduction pathways [33]. In case of DLBCL, especially Epstein-Barr virus- (EBV-) positive mediastinal type, PD-L1 is overexpressed by signaling of activator protein 1 and very probably EBV latent membrane protein 1 [34]. PD-L1 overexpression is observed also in FL or even in anaplastic cell lymphoma (ALCL) [35].

Preclinical studies suggested that tumor cells in mantle cell lymphoma (MCL) can evade the immune antitumor response by several microenvironmental factors, including T cells which are able to inhibit cytokine CD4+CD25− production by interaction between PD-1 and PD-L1. It was observed that PD-L1 expression on MCL cells inhibits T cell-mediated tumor cytotoxicity and their specific antitumor response [36, 37].

As the biology of PD-1 signaling pathway, PD-1 expression can be best tested in the microenvironment of lymphoid malignancies. PD-1 expression in tumor-infiltrating lymphocytes (TILs) has been reported in follicular lymphoma (FL) and nodular lymphocyte predominant HL [38, 39]. Since both malignant lymphoma cells arise from germinal-center B cells, it is not surprise that their microenvironments mimic their normal counterparts. What is more, the PD-1-positive TILs in lymphomas may indicate the origin of cells due to PD-1-positive TILs in DLBCL, and FL is connected with a good prognosis [38]. It is different than in solid tumors, where the presence of PD-1-positive TILs is connected with worse prognosis [40].

Still, little is known about the expression of CTLA-4 in the human tissue. So far, it was reported that CD80 and CD86, physiological ligands for CTLA-4 expression, may be seen in patients with T cell lymphomas in the cell of dendritic system, a subset of germinal-center B cells and B immunoblasts in lymphoma nodes and Reed-Sternberg cells. It was already observed in patients with peripheral T cell lymphoma, mycosis fungoides, and Sézary syndrome [41, 42]. CTLA4-CD28 rearrangement is also present in a subset of patients with angioimmunoblastic T cell lymphoma, extranodal NK/T cell lymphoma, peripheral T cell lymphoma, not otherwise specified, Sézary syndrome, and adult T cell leukemia/lymphoma [43, 44]. The rearrangement generates a fusion protein including the extracellular and transmembrane domains of CTLA4 and the cytoplasmic domain of CD28, which mediates activating T cell signals via AKT and MAPK pathways [45]. Moreover, CTLA-4 has emerged as key target of checkpoint inhibition demonstrating high activity particularly in heavily pretreated relapsed/refractory HL and some types of NHLs.

## 4. Immunobiology of TIM-3 and LAG-3 in Lymphoma

The clinical success of targeting PD-1 and CTLA-4 receives more attention on lymphocyte activation gene-3 (LAG-3, CD223) and T cell immunoglobulin and mucin domain-containing protein-3 (TIM-3). The LAG-3 is expressed in activated T and B cells, NK cells, and plasmacytoid dendritic cells [46]. Although the exact mechanism of action is still unknown, LAG-3 is a negative regulator in CD4 and CD8 T cell expansion *in vitro* as well as *in vivo* [47]. LAG-3 and PD-1 have been reported to be coexpressed both in TILs in tumor mouse models and human tissue [48]. It suggested to have a similar role to PD-1. Moreover, the inhibition of PD-1 as well as LAG-3 showed augmented anticancer activity of CD8+ T cells as compared with targeting each of them [49].

TIM-3 expression is present in cytotoxic T cells, T helper 1 cells, regulatory T cells, NK cells, monocytes, and dendritic cells. Similar to LAG-3, the coexpression of TIM-3 and PD-1 was observed in CD8+ TILs [50, 51]. It was observed that high expression of TIM-3 is present in FL, and it correlates with higher histological grade and serum lactate dehydrogenase (LDH) concentration [52, 53]. Yang et al. in his work reported that IL-12 therapy increases TIM-3 expression by FL TILs [52]. Moreover, they observed that TIM-3 positive TILs exhibit functional impairment when stimulated with PMA/ionomycin (PMA/ion). Last but not least, they reported that patients with a higher number of CD4+TIM-3+ T cells in FL cellular suspensions showed a worse prognosis comparing with FL patients with a lower number of CD4+TIM-3+ T cells. 2 y PFS rate was 82% versus 100%, respectively.

## 5. Clinical Experiences on Immune Checkpoint Blockade in HL

### 5.1. PD-1 Blockade

PD-1 and its ligands have been shown to play a significant role in evasion of cancer cells from the immune system. In 2016, the U.S. FDA approved anti-PD-1 inhibitors for treatment of non-small-cell lung carcinoma and recently expanded the use of immunotherapy for metastatic urothelial cell carcinoma and relapsed cHL as the first hematological indication. PD-1/PD-L1 inhibitors have been successful in the therapy of cHL, which typically exhibits an overexpression of PD-1 ligands (PD-L1, PD-L2) due to near-universal genetic changes in chromosome locus 9p24.1 [54]. In a work by Roemer et al. in series of 108 biopsy specimens from newly diagnosed cHL patients, 105 (97%) had increased expression of PD-L1 and/or PD-L2 [55]. Moreover, authors observed an association between PD-L1 protein expression and relative genetic alterations in this series. For patients with 9p24.1 amplification, progression-free survival (PFS) was shorter, and the incidence of 9p24.1 amplification was more frequent along patients with advanced stages. These results may indicate a possible genetic dependence upon PD-1 signaling in cHL.

PD-1 antibody in monotherapy in patients with HL diagnosis has already shown safety and effectiveness in clinical studies (Table 2). In a phase I study, nivolumab in 23 R/R HL that had already been heavily treated demonstrated an objective response in 87% (20 patients) of patients [56]. CR was observed in 17%, partial response (PR) in 70%, and stable disease (SD) in 13%. The rate of PFS after 2 years was 86%. Drug-related side effects of any grade and of grade 3 occurred in 78% and 22% of patients, respectively. In a CheckMate phase II clinical trial, 80 patients with R/R cHL after ASCT and brentuximab vedotin were treated with nivolumab [57]. An objective response rate in this heavily treated patients was of 66% with 9% CR and 58% PR. Safety profile was acceptable with the most common drug-related grade 3 or 4 AE which is neutropenia (5%) and increased lipase concentrations (5%).

Long-term follow-up from two mentioned studies demonstrated acute graft versus host disease (GVHD) in 82% of cHL patients treated with nivolumab who had allo-HSCT [58]. AGVHD is a major cause of morbidity and mortality, affecting approximately 60% of allo-HSCT recipients, who were not treated with PD-1 inhibitor. It suggests that anti-PD-1 treatment before allo-HSCT might be responsible for higher risk of immune-related complications after transplantation procedure. AE of grades 2–4 was observed in 10 patients (59%) and grades 3-4 in 5 patients (29%). The organs involved were skin, gut, liver, and lung. Two patients had hyperacute GVHD (onset ≤ 14 days after allo-HSCT), and one patient died as a result of multiorgan GVHD.

Other clinical studies for R/R cHL that are recently recruiting participants are NCT02572167 (brentuximab vedotin combined with nivolumab), NCT01896999 (brentuximab vedotin and nivolumab with or without ipilimumab), and NCT02940301 (ibrutinib and nivolumab). Preliminary data from the phase I trial of nivolumab plus ipilimumab were presented at ASH 2016 [59]. Among 31 patients with R/R HL, an overall response rate (ORR) was 74% with 19% CR and 55% PR. Safety profile was tolerable with the most common side effect, fatigue. 29% of patients had a drug-related AE of grade ≥ 3. There were no deaths observed due to treatment. Also, data from NCT01896999 study were presented at ASH 2016 [60]. 8 R/R HL patients treated with brentuximab, ipilimumab, and nivolumab had ORR of 100%. CR observed in this study was 63%. The PFS to date was 100% with a median follow-up of 0.3 years. In this heavily pretreated group, therapy was well tolerated with one pneumonitis grade 3, with grade 3 dyspnea and hypoxia, and with grade 3 typhilits.

Another PD-1 inhibitor that demonstrated high efficacy in R/R cHL patients is pemolizumab. In a phase Ib clinical trial, 31 R/R cHL patients were included [61]. The ORR was 58%, with 19% CR, 12% PR, and 23% SD. Median PFS was 11.4 and median OS was not reached. Safety profile was tolerable with only 3 patients (10%) who discontinued treatment due to drug-related toxicity. In a multicenter phase II study, pembrolizumab was evaluated in 3 cohorts of patients with R/R cHL (after ASCT and brentuximab vedotin, ineligibility for ASCT due to chemoresistance and brentuximab vedotin therapy failure, and after ASCT but not treated with brentuximab vedotin) [62]. Among 210 R/R HL, the ORR was 67% in cohort 1, 65% in cohort 2, and 68% in cohort 3. The CR was 29% in cohort 1, 25% in cohort 2, and 22% in cohort 3. The most common grade 3 and 4 side effects were myelosuppression and diarrhea. This preliminary data suggested significant clinical efficacy of pembrolizumab in all three groups, including refractory patients.

In the study by Kwong et al., two patients with refractory cHL were treated with pembrolizumab at a low dose [63]. Both patients were after brentuximab vedotin treatment, one relapsed after ASCT. Both patients had advanced stage of the disease (III and IV clinical stage). After pembrolizumab therapy, both of them achieved CR and were stable for >20 weeks. Moreover, no side effects were observed. In this study, patients received a lower dose of pembrolizumab; however, it had still therapeutic activity and safe profile. In another study, also two patients with relapsed cHL were treated with pembrolizumab but in standard dose [64]. Both patients were heavily pretreated and failed after many lines of treatment including allo-HSCT. The response to single-agent pembrolizumab treatment was very good. One patient achieved CR and the second PR. Both of them remain on treatment without evidence of disease progression. What is more, in this study, no GVHD was observed. It is the first data that describe the safe and successful use of pembrolizumab in patients after allo-HSCT.

PD-1 inhibitors in cHL are currently in clinical development. There are planned phase III study of nivolumab monotherapy for cHL and a phase III study comparing pembrolizumab with brentuximab vedotin. Moreover, there will be studies evaluating therapy with anti-PD-1 drugs earlier in the natural history of cHL. Data from trials mentioned above caused accelerated FDA approval for nivolumab in May 2016 in patients with cHL diagnosis who are refractory to ASCT and brentuximab vedotin without a confirmatory phase III clinical trial [65]. Also, pembrolizumab has been approved by the FDA in May 2017 for treatment of cHL patients whose disease is refractory or has relapsed after at least three prior therapies [66]. The approval marks the first time that a PD-1 inhibitor has been indicated for adults and for children. There are still some doubts about allo-HSCT following PD-1 inhibitor treatment because of potential higher risk of GVHD.

In one phase I study, the only participating MCL patient did not respond to ipilimumab [67]. Another study, however, showed MCL patient who respond to ipilimumab prior to relapse after allo-HSCT [68]. Finally, another four MCL patients did not respond to nivolumab [69]. Probably, these patients, but also some T cell lymphoma patients, might benefit from combination with other treatments in the near future.

### 5.2. CTLA-4 Blockade

CTLA-4 inhibitor ipilimumab was evaluated in a phase I clinical study by Diefenbach et al. presented at the 2015 American Society of Hematology (ASH) Annual Meeting [70]. In 23 relapsed and refractory cHL patients, ipilimumab was given with brentuximab vedotin. In this preliminary study, this drug combination was well tolerated, with only manageable immune-related side effect. Moreover, among 12 evaluable patients, the ORR was 67%, and the CR rate was 42%. Data from this clinical trial shows that this drug combination has promising efficacy and needs further investigation.

Ipilimumab was investigated in two phase I studies in hematologic malignancies after allo-HSCT. In first study by Bashey et al., 29 patients with advanced hematologic diseases (14 with HL) were treated with ipilimumab [68]. Objective responses were observed in 3 patients out of 18 treated with dose > 1.0 mg/kg (17%), and 2 durable CR were observed in patients with HL diagnosis. Ipilimumab was generally well tolerated with grade 3/4 AE occurred in 4 patients (13%). Preliminary data of the expansion cohort of this study were reported at the 2015 ASH Annual Meeting [71]. 28 patients with R/R hematologic malignancies, including 7 with HL, were evaluated. Among 21 patients, the ORR was 33%; 1 patient with HL achieved PR. These two clinical trials demonstrated that CTLA-4 inhibitor has safe profile in patients after allo-HSCT procedure.

## 6. Clinical Trials of Immune Checkpoint Blockade in Non-Hodgkin Lymphoma

### 6.1. PD-1 Blockade

In contrast to cHL, only 25% of patients with DLBCL express PD-1 [72]. The only exception is primary mediastinal B cell lymphoma (PMBCL) where, like in cHL, we can often observe high expression of PD-1 pathway [73]. In phase Ib multicenter study, pembrolizumab was administered to relapsed and refractory PMBCL [74]. 19 patients were enrolled to this study; 9 were evaluated for response. The ORR was 44%, with 11% CR (1 out of 9) and 33% PR (3 out of 9). Among all the treated patients, there were no grade 3/4 AEs. As pembrolizumab had a tolerable safety profile and a promising efficacy in relapsed and refractory PMBCL patients, a multicenter phase II study is recently ongoing [75].

Not surprisingly, the results of treatment with checkpoint inhibitors in NHL are not such spectacular as in cHL and PMBL. The clinical trials are shown in Table 3. In the multicenter phase I clinical trial, 17 patients with different types of advanced hematologic malignancies were treated with pembrolizumab [76]. The ORR for all the groups was 33% with one CR observed in patient with FL. Moreover, according to the study, the drug was not only effective but also well tolerated. Due to promising results of phase I study, a large multicenter phase II study was designed [77]. 66 patients with DLBCL diagnosis and after ASCT procedure were enrolled to this study. The ORR was 51% with 34% of CR, 17% PR, and 37% SD. The 16-month PFS was 70% and OS was not reached. It is the first clinical trial that showed efficacy of PD-1 inhibitors in patients with relapsed and refractory DLBCL.

Also, nivolumab was evaluated in patients with relapsed and refractory B cell NHL and in a phase I study, with 54 patients with NHL including 10 FL, 11 DLBCL, 10 other B cell lymphomas, 13 peripheral T cell lymphoma (PTCL), and 5 other T cell lymphomas [69]. The highest ORR was observed among FL patients, 40% (1 CR, 2 PR), and next in DLBCL, 36% (2 CR, 2 PR). Drug was well tolerated; AEs occurred in 66%; majority of them were grades 1 and 2. Recently, phase II studies are ongoing in patients with NHL diagnosis.

While there are still patients who do not benefit from single-agent checkpoint inhibitor treatment, new combinations of drugs are currently under investigation in NHL patients. Checkpoint inhibitors are combined in various ways with standard cytotoxic drugs, monoclonal antibodies, and novel immunomodulating agents. One example is the combination of PD-1 blockade with ibrutinib, a Bruton's tyrosine kinase inhibitor, which targets B cell receptor signaling pathway [78]. The therapeutic cytotoxic activity of checkpoint inhibitor is enhanced by ibrutinib making this combination very promising.

### 6.2. CTLA-4 Blockade

Although the exact role of the CTLA-4 blockade in DLBCL is still unknown, ipilimumab was one of the first agents evaluated in this diagnosis. In the phase I study of ipilimumab in patients with R/R B cell NHL, 18 patients were enrolled [67]. Two patients had clinical responses; the ORR was low, only 11%. One with DLBCL achieved a durable CR lasting >31 months, and one with FL had a PR lasting 19 months. Ipilimumab was well tolerated, with typical side effects like headache, diarrhea, anorexia, abdominal pain, easy fatigue, and myelosuppression. In a work by Sekulic et al., one patient with advanced Sezary syndrome was treated with ipilimumab [44]. The patient demonstrated a marked clinical response including 50% reduction in erythema, 75% size reduction of dermal and subcutaneous tumors with 50% size reduction of lower leg ulcers, and self-reported decrease in itching. Moreover, no drug-related toxicity was observed. The patient reported higher energy level that enables everyday life activities. Unfortunately, 6 weeks after the end of treatment, the disease rapidly progressed. Patient died 3 months later.

## 7. Side Effects of Checkpoint Inhibitors

Like every novel agents, checkpoint blockade has some side effects. The most commonly observed drug-related AEs are gastrointestinal, hepatic, dermatologic, and endocrine events. If AE is grade 2, doctors usually recommend to withhold the therapy temporarily, but if AE grade 3 or higher occurs, checkpoint inhibitor must be stopped. It was observed that side effects after PD-1 inhibitors occur more seldom than after CTLA-4 inhibitors [79]. In a study by Horvat et al., 298 patients with melanoma diagnosis had ipilimumab therapy [80]. The side effects were observed in 85% of patients, while only 38% were grade 3 or higher. The most commonly reported AEs were diarrhea, hepatotoxicity, dermatitis, hypophysitis, and uveitis. 33% of patients were treated with systemic corticosteroids due to drug-related side effects, but it did not affect OS. In another study with 576 melanoma patients, nivolumab was administered [81]. Among all patients, 71% had AE, but only 10% was grade 3 and higher. The most common drug-related AEs were fatigue (25%), pruritus (17%), diarrhea (13%), and rash (13%). There were no drug-related deaths. The ORRs were similar in patients who received and did not receive treatment due to side effects. In conclusion, treatment-related side effects with checkpoint inhibitor monotherapy were mostly easily manageable and did not affect ORR.

## 8. Conclusions

Immunological checkpoint inhibitors have emerged as a successful therapeutic option at first for the therapy of different solid cancers, and now it is rapidly exploring in hematologic diseases as well. Early data from clinical studies of anti-PD-1 monoclonal antibodies have demonstrated that they are both highly effective and have satisfactory safety profile, especially in patients with cHL. What is important among cHL patients, the response rates with checkpoint blockade is high even in patients with highly refractory disease after multiple lines of prior therapies. The next step is to provide a rationale for targeting multiple immune checkpoints to enhance antitumor immunity, better understanding of prognostic factors, and mechanisms of action and to find the optimal combinations of drugs to reach better effect. The future in the treatment of cHL will possibly be a combination of checkpoint inhibitors with other novel therapies in the hope of higher response rates that can change the course of this disease.

## Figures and Tables

**Table 1:** Immune checkpoint inhibitors (monoclonal antibodies) that target PD-1, PD-L1, or CTLA-4 used in different types of cancer.

Immune checkpoint inhibitor	Target	Malignancy
Pembrolizumab (Keytruda) and nivolumab (Opdivo)	PD-1	Melanoma of the skin, non-small-cell lung cancer, kidney cancer, bladder cancer, head and neck cancers, and Hodgkin's lymphoma
Atezolizumab (Tecentriq), avelumab (Bavencio), and durvalumab (Imfinzi)	PD-L1	Bladder cancer, non-small-cell lung cancer, and Merkel cell carcinoma
Ipilimumab (Yervoy)	CTLA-4	Skin melanoma

**Table 2:** Clinical efficacy of checkpoint inhibitors in relapsed/refractory HL.

Drug	Ph	Target	*N*	ORR (%)	CR (%)	PR (%)	SD (%)	OS	PFS	Ref
Nivolumab	I	Anti-PD-1	23	87	17	70	13		2 years, 86%	10
Nivolumab	II	Anti-PD-1	80	66	9	58	NR	6 m 77%	6 m, 77%	11
Nivolumab+	I	Anti-PD-1	31	74	19	55	10	NR	NR	13
Nivolumab + BV	I	Anti-PD-1	10	100	63			NR	4 m, 100%	14
Pemolizumab	Ib	Anti-PD-1	31	58	19	12	23	NR	11.4	15
Pemolizumab	II	Anti-PD-1	210	65–68	22–29					16
Ipilimumab	I	Anti-CTLA-4	12	67	42				0.74 years	19

HL: Hodgkin lymphoma; Ph: phase; *N*: number of patients; m: month; ORR: overall response rate; CR: complete response; PR: partial response; SD: stable disease; OS: overall response; PFS: progression-free survival; Ref: reference; R/R: relapsed and refractory; BV: brentuximab vedotin; NR: not reached.

**Table 3:** Clinical efficacy of checkpoint inhibitors in other hematologic malignancies.

Drug	Ph	Target	Disease	*N*	ORR (%)	CR (%)	PR (%)	SD (%)	OS	PFS	Ref
Pembrolizumab	I	Anti-PD-1	PMBCL	19	44	11	33		NR		24
Pembrolizumab	II	Anti-PD-1	DLBCL	66	51	34	17	37	NR	16 m, 70%	27
Ipilimumab	I	Anti-CTLA-4	NHL	18	11	5.5	5.5				30

Ph: phase; *N*: number of patients; m: month; ORR: overall response rate; CR: complete response; PR: partial response; SD: stable disease; OS: overall response; PFS: progression-free survival; Ref: reference; BV: brentuximab vedotin; NR: not reached.

## Abbreviations

AE: Adverse event
ALCL: Anaplastic cell lymphoma
Allo-HSCT: Allogeneic hematopoietic stem-cell transplant
APC: Antigen-presenting cell
ASCT: Autologous stem-cell transplantation
cHL: Classical Hodgkin lymphoma
CR: Complete remission,
CTLA-4: Cytotoxic T lymphocyte-associate protein-4
DLBCL: Diffuse large B cell lymphoma
FL: Follicular lymphoma
HL: Hodgkin lymphoma
MCL: Mantle cell lymphoma
NHL: Non-Hodgkin lymphoma
OS: Overall survival
PD: Progressive disease
PD-1: Programmed cell death 1 receptor
PDL-1: Programmed cell death 1 receptor ligand
PFS: Progression-free survival
PMBCL: Primary mediastinal B cell lymphoma
PR: Partial response
R/R: Resistant/relapsed
TNFR: Tumor necrosis factor receptor
Tregs: T regulatory cells.
